# Supplementary material for: Improved procedures and computer programs for equivalence assessment of correlation coefficients
Source: PLoS One. 2021 May 28;16(5):e0252323. doi: 10.1371/journal.pone.0252323 (PMC8162672; doi:10.1371/journal.pone.0252323)
Supplement: S1 File — (DOCX) [file pone.0252323.s001.docx]

File R1

R program for computing the critical intervals of the correlation equivalence procedures

function (){

#USER SPECIFICATIONS PORTION

alpha=0.05 #type I error rate

n=25 #sample sizes

rhobl=0 #equivalence bounds

rhobu=0.2

#END OF SPECIFICATIONS PORTION

rho=rhobl

za=qnorm(1-alpha)

print(c("rhobl,rhobu,alpha,n"))

print(c(rhobl,rhobu,alpha,n))

ealufun=function(){

tolb=1e-10

d=0.05

alphat=0

loopm=1e03

loop1=0

avarl=((1-rhobl^2)^2)/(n-3)

avaru=((1-rhobu^2)^2)/(n-3)

astdl=sqrt(avarl)

astdu=sqrt(avaru)

c1=(rhobl+rhobu)/2

while (alphat<alpha & loop1<loopm){

c1=c1-d

c1cdfu=pnorm(c1,rhobu,astdu)

c2=qnorm(c1cdfu+alpha,rhobu,astdu)

c1cdfl=pnorm(c1,rhobl,astdl)

c2cdfl=pnorm(c2,rhobl,astdl)

alphat=c2cdfl-c1cdfl}

c1l=c1

c1u=c1+d

loop=0

dalpha=-10

while (dalpha<0 | dalpha>tolb & loop<loopm){

c1=(c1l+c1u)/2

c1cdfu=pnorm(c1,rhobu,astdu)

c2=qnorm(c1cdfu+alpha,rhobu,astdu)

c1cdfl=pnorm(c1,rhobl,astdl)

c2cdfl=pnorm(c2,rhobl,astdl)

alphat=c2cdfl-c1cdfl

dalpha=alphat-alpha

loop=loop+1

if (dalpha<0) {c1u=c1} else {c1l=c1} }

real=c1

reau=c2

return(c(real,reau))}

eflufun=function(){

tolb=1e-10

d=0.05

alphat=0

loopm=1e03

loop1=0

taubl=log((1+rhobl)/(1-rhobl))/2

taubu=log((1+rhobu)/(1-rhobu))/2

tauvar=1/(n-3)

taustd=sqrt(tauvar)

c1=(taubl+taubu)/2

while (alphat<alpha & loop1<loopm){

c1=c1-d

c1cdfu=pnorm(c1,taubu,taustd)

c2=qnorm(c1cdfu+alpha,taubu,taustd)

c1cdfl=pnorm(c1,taubl,taustd)

c2cdfl=pnorm(c2,taubl,taustd)

alphat=c2cdfl-c1cdfl}

c1l=c1

c1u=c1+d

loop=0

dalpha=-10

while (dalpha<0 | dalpha>tolb & loop<loopm){

c1=(c1l+c1u)/2

c1cdfu=pnorm(c1,taubu,taustd)

c2=qnorm(c1cdfu+alpha,taubu,taustd)

c1cdfl=pnorm(c1,taubl,taustd)

c2cdfl=pnorm(c2,taubl,taustd)

alphat=c2cdfl-c1cdfl

dalpha=alphat-alpha

loop=loop+1

if (dalpha<0) {c1u=c1} else {c1l=c1} }

tauel=c1

taueu=c2

refl=(exp(2*tauel)-1)/(exp(2*tauel)+1)

refu=(exp(2*taueu)-1)/(exp(2*taueu)+1)

return(c(refl,refu)) }

rea=ealufun()

print(c("EQUT-R: ",round(rea,4)))

ref=eflufun()

print(c("EQUT-ZETA_HAT: ",round(ref,4))) }

File R2

R program for computing the power of the correlation equivalence procedures

function (){

#USER SPECIFICATIONS PORTION

alpha=0.05 #type I error rate

n=834 #sample sizes

rho=0.1 #true correlation

rhobl=0 #equivalence bounds

rhobu=0.2

#END OF SPECIFICATIONS PORTION

za=qnorm(1-alpha)

print(c("rho,rhobl,rhobu,alpha,n"))

print(c(rho,rhobl,rhobu,alpha,n))

ealufun=function(){

tolb=1e-10

d=0.05

alphat=0

loopm=1e03

loop1=0

avarl=((1-rhobl^2)^2)/(n-3)

avaru=((1-rhobu^2)^2)/(n-3)

astdl=sqrt(avarl)

astdu=sqrt(avaru)

c1=(rhobl+rhobu)/2

while (alphat<alpha & loop1<loopm){

c1=c1-d

c1cdfu=pnorm(c1,rhobu,astdu)

c2=qnorm(c1cdfu+alpha,rhobu,astdu)

c1cdfl=pnorm(c1,rhobl,astdl)

c2cdfl=pnorm(c2,rhobl,astdl)

alphat=c2cdfl-c1cdfl}

c1l=c1

c1u=c1+d

loop=0

dalpha=-10

while (dalpha<0 | dalpha>tolb & loop<loopm){

c1=(c1l+c1u)/2

c1cdfu=pnorm(c1,rhobu,astdu)

c2=qnorm(c1cdfu+alpha,rhobu,astdu)

c1cdfl=pnorm(c1,rhobl,astdl)

c2cdfl=pnorm(c2,rhobl,astdl)

alphat=c2cdfl-c1cdfl

dalpha=alphat-alpha

loop=loop+1

if (dalpha<0) {c1u=c1} else {c1l=c1} }

real=c1

reau=c2

return(c(real,reau))}

eflufun=function(){

tolb=1e-10

d=0.05

alphat=0

loopm=1e03

loop1=0

taubl=log((1+rhobl)/(1-rhobl))/2

taubu=log((1+rhobu)/(1-rhobu))/2

tauvar=1/(n-3)

taustd=sqrt(tauvar)

c1=(taubl+taubu)/2

while (alphat<alpha & loop1<loopm){

c1=c1-d

c1cdfu=pnorm(c1,taubu,taustd)

c2=qnorm(c1cdfu+alpha,taubu,taustd)

c1cdfl=pnorm(c1,taubl,taustd)

c2cdfl=pnorm(c2,taubl,taustd)

alphat=c2cdfl-c1cdfl}

c1l=c1

c1u=c1+d

loop=0

dalpha=-10

while (dalpha<0 | dalpha>tolb & loop<loopm){

c1=(c1l+c1u)/2

c1cdfu=pnorm(c1,taubu,taustd)

c2=qnorm(c1cdfu+alpha,taubu,taustd)

c1cdfl=pnorm(c1,taubl,taustd)

c2cdfl=pnorm(c2,taubl,taustd)

alphat=c2cdfl-c1cdfl

dalpha=alphat-alpha

loop=loop+1

if (dalpha<0) {c1u=c1} else {c1l=c1} }

tauel=c1

taueu=c2

refl=(exp(2*tauel)-1)/(exp(2*tauel)+1)

refu=(exp(2*taueu)-1)/(exp(2*taueu)+1)

return(c(tauel,taueu,refl,refu)) }

eapowerfun=function(){

rea=ealufun()

avar=((1-rho^2)^2)/(n-3)

astd=sqrt(avar)

zeal=(rea[1]-rho)/astd

zeau=(rea[2]-rho)/astd

powerea=pnorm(zeau)-pnorm(zeal)}

efpowerfun=function(){

ref=eflufun()

taurho=log((1+rho)/(1-rho))/2

tauvar=1/(n-3)

taustd=sqrt(tauvar)

zefl=(ref[1]-taurho)/taustd

zefu=(ref[2]-taurho)/taustd

poweref=pnorm(zefu)-pnorm(zefl)}

powerea=eapowerfun()

print(c("EQUT-R: ",round(powerea,4)))

poweref=efpowerfun()

print(c("EQUT-ZETA_HAT: ",round(poweref,4))) }

FILE R3

R program for computing the sample sizes of the correlation equivalence procedures

function (){

#USER SPECIFICATIONS PORTION

alpha=0.05 #type I error rate

power=0.80 #nominal power

rho=0.1 #true correlation

rhobl=0 #equivalence bounds

rhobu=0.2

#END OF SPECIFICATIONS PORTION

za=qnorm(1-alpha)

print(c("rho,rhobl,rhobu,alpha,power"))

print(c(rho,rhobl,rhobu,alpha,power))

ealufun=function(){

tolb=1e-10

d=0.05

alphat=0

loopm=1e03

loop1=0

avarl=((1-rhobl^2)^2)/(n-3)

avaru=((1-rhobu^2)^2)/(n-3)

astdl=sqrt(avarl)

astdu=sqrt(avaru)

c1=(rhobl+rhobu)/2

while (alphat<alpha & loop1<loopm){

c1=c1-d

c1cdfu=pnorm(c1,rhobu,astdu)

c2=qnorm(c1cdfu+alpha,rhobu,astdu)

c1cdfl=pnorm(c1,rhobl,astdl)

c2cdfl=pnorm(c2,rhobl,astdl)

alphat=c2cdfl-c1cdfl}

c1l=c1

c1u=c1+d

loop=0

dalpha=-10

while (dalpha<0 | dalpha>tolb & loop<loopm){

c1=(c1l+c1u)/2

c1cdfu=pnorm(c1,rhobu,astdu)

c2=qnorm(c1cdfu+alpha,rhobu,astdu)

c1cdfl=pnorm(c1,rhobl,astdl)

c2cdfl=pnorm(c2,rhobl,astdl)

alphat=c2cdfl-c1cdfl

dalpha=alphat-alpha

loop=loop+1

if (dalpha<0) {c1u=c1} else {c1l=c1} }

real=c1

reau=c2

return(c(real,reau))}

eflufun=function(){

tolb=1e-10

d=0.05

alphat=0

loopm=1e03

loop1=0

taubl=log((1+rhobl)/(1-rhobl))/2

taubu=log((1+rhobu)/(1-rhobu))/2

tauvar=1/(n-3)

taustd=sqrt(tauvar)

c1=(taubl+taubu)/2

while (alphat<alpha & loop1<loopm){

c1=c1-d

c1cdfu=pnorm(c1,taubu,taustd)

c2=qnorm(c1cdfu+alpha,taubu,taustd)

c1cdfl=pnorm(c1,taubl,taustd)

c2cdfl=pnorm(c2,taubl,taustd)

alphat=c2cdfl-c1cdfl}

c1l=c1

c1u=c1+d

loop=0

dalpha=-10

while (dalpha<0 | dalpha>tolb & loop<loopm){

c1=(c1l+c1u)/2

c1cdfu=pnorm(c1,taubu,taustd)

c2=qnorm(c1cdfu+alpha,taubu,taustd)

c1cdfl=pnorm(c1,taubl,taustd)

c2cdfl=pnorm(c2,taubl,taustd)

alphat=c2cdfl-c1cdfl

dalpha=alphat-alpha

loop=loop+1

if (dalpha<0) {c1u=c1} else {c1l=c1} }

tauel=c1

taueu=c2

refl=(exp(2*tauel)-1)/(exp(2*tauel)+1)

refu=(exp(2*taueu)-1)/(exp(2*taueu)+1)

return(c(tauel,taueu,refl,refu)) }

eapowerfun=function(){

rea=ealufun()

avar=((1-rho^2)^2)/(n-3)

astd=sqrt(avar)

zeal=(rea[1]-rho)/astd

zeau=(rea[2]-rho)/astd

powerea=pnorm(zeau)-pnorm(zeal)}

efpowerfun=function(){

ref=eflufun()

taurho=log((1+rho)/(1-rho))/2

tauvar=1/(n-3)

taustd=sqrt(tauvar)

zefl=(ref[1]-taurho)/taustd

zefu=(ref[2]-taurho)/taustd

poweref=pnorm(zefu)-pnorm(zefl)}

n=20

powerea=0

while (powerea<power){

n=n+1

powerea=eapowerfun()}

print(c("EQUT-R: ",n,round(powerea,4)))

n=20

poweref=0

while (poweref<power){

n=n+1

poweref=efpowerfun()}

print(c("EQUT-ZETA_HAT: ",n,round(poweref,4))) }
