# Supplementary material for: Improved procedures and computer programs for equivalence assessment of correlation coefficients
Source: PLoS One. 2021 May 28;16(5):e0252323. doi: 10.1371/journal.pone.0252323 (PMC8162672; doi:10.1371/journal.pone.0252323)
Supplement: S2 File — (DOCX) [file pone.0252323.s002.docx]

FILE S1

SAS/IML program for computing the critical intervals of the correlation equivalence procedures

PROC IML;

*USER SPECIFICATION PORTION;

*TYPE I ERROR;ALPHA=0.05;

*SAMPLE SIZES;N=25;

*EQUIVALENCE BOUNDS;RHOBL=0;RHOBU=0.2;

*END OF SPECIFICATION PORTION;

RHO=RHOBU;

ZA=PROBIT(1-ALPHA);

PRINT RHOBL RHOBU ALPHA N;

START EALUFUN;

TOLB=1E-10;D=0.05;

ALPHAT=0;LOOPM=1E03;LOOP1=0;

AVARL=((1-RHOBL##2)##2)/(N-3);ASTDL=SQRT(AVARL);

AVARU=((1-RHOBU##2)##2)/(N-3);ASTDU=SQRT(AVARU);

C1=(RHOBL+RHOBU)/2;

DO WHILE(ALPHAT<ALPHA & LOOP1<LOOPM);

C1=C1-D;

C1CDFU=CDF('NORMAL',C1,RHOBU,ASTDU);

C2=QUANTILE('NORMAL',C1CDFU+ALPHA,RHOBU,ASTDU);

C1CDFL=CDF('NORMAL',C1,RHOBL,ASTDL);

C2CDFL=CDF('NORMAL',C2,RHOBL,ASTDL);

ALPHAT=C2CDFL-C1CDFL;END;

C1L=C1;C1U=C1+D;LOOP=0;DALPHA=-10;

DO WHILE(DALPHA<0 | DALPHA>TOLB & LOOP<LOOPM);

C1=(C1L+C1U)/2;

C1CDFU=CDF('NORMAL',C1,RHOBU,ASTDU);

C2=QUANTILE('NORMAL',C1CDFU+ALPHA,RHOBU,ASTDU);

C1CDFL=CDF('NORMAL',C1,RHOBL,ASTDL);

C2CDFL=CDF('NORMAL',C2,RHOBL,ASTDL);

ALPHAT=C2CDFL-C1CDFL;

DALPHA=ALPHAT-ALPHA;LOOP=LOOP+1;

IF DALPHA<0 THEN C1U=C1;ELSE C1L=C1;END;

REAL=C1;REAU=C2;FINISH;

START EFLUFUN;

TOLB=1E-10;D=0.05;

ALPHAT=0;LOOPM=1E03;LOOP1=0;

TAUBL=LOG((1+RHOBL)/(1-RHOBL))/2;

TAUBU=LOG((1+RHOBU)/(1-RHOBU))/2;

TAUVAR=1/(N-3);TAUSTD=SQRT(TAUVAR);

C1=(TAUBL+TAUBU)/2;

DO WHILE(ALPHAT<ALPHA & LOOP1<LOOPM);

C1=C1-D;

C1CDFU=CDF('NORMAL',C1,TAUBU,TAUSTD);

C2=QUANTILE('NORMAL',C1CDFU+ALPHA,TAUBU,TAUSTD);

C1CDFL=CDF('NORMAL',C1,TAUBL,TAUSTD);

C2CDFL=CDF('NORMAL',C2,TAUBL,TAUSTD);

ALPHAT=C2CDFL-C1CDFL;END;

C1L=C1;C1U=C1+D;LOOP=0;DALPHA=-10;

DO WHILE(DALPHA<0 | DALPHA>TOLB & LOOP<LOOPM);

C1=(C1L+C1U)/2;

C1CDFU=CDF('NORMAL',C1,TAUBU,TAUSTD);

C2=QUANTILE('NORMAL',C1CDFU+ALPHA,TAUBU,TAUSTD);

C1CDFL=CDF('NORMAL',C1,TAUBL,TAUSTD);

C2CDFL=CDF('NORMAL',C2,TAUBL,TAUSTD);

ALPHAT=C2CDFL-C1CDFL;

DALPHA=ALPHAT-ALPHA;LOOP=LOOP+1;

IF DALPHA<0 THEN C1U=C1;ELSE C1L=C1;END;

TAUEL=C1;TAUEU=C2;

REFL=(EXP(2#TAUEL)-1)/(EXP(2#TAUEL)+1);

REFU=(EXP(2#TAUEU)-1)/(EXP(2#TAUEU)+1);FINISH;

RUN EALUFUN;RUN EFLUFUN;

PRINT 'EQUT-R ' REAL[FORMAT=8.4] REAU[FORMAT=8.4];

PRINT 'EQUT-ZETA_HAT' REFL[FORMAT=8.4] REFU[FORMAT=8.4];

QUIT;

FILE S2

SAS/IML program for computing the power of the correlation equivalence procedures

PROC IML;

*USER SPECIFICATION PORTION;

*TYPE I ERROR;ALPHA=0.05;

*SAMPLE SIZES;N=834;

*TRUE CORRELATION;RHO=0.1;

*EQUIVALENCE BOUNDS;RHOBL=0;RHOBU=0.2;

*END OF SPECIFICATION PORTION;

ZA=PROBIT(1-ALPHA);

PRINT RHO RHOBL RHOBU ALPHA N;

START EALUFUN;

TOLB=1E-10;D=0.05;

ALPHAT=0;LOOPM=1E03;LOOP1=0;

AVARL=((1-RHOBL##2)##2)/(N-3);ASTDL=SQRT(AVARL);

AVARU=((1-RHOBU##2)##2)/(N-3);ASTDU=SQRT(AVARU);

C1=(RHOBL+RHOBU)/2;

DO WHILE(ALPHAT<ALPHA & LOOP1<LOOPM);

C1=C1-D;

C1CDFU=CDF('NORMAL',C1,RHOBU,ASTDU);

C2=QUANTILE('NORMAL',C1CDFU+ALPHA,RHOBU,ASTDU);

C1CDFL=CDF('NORMAL',C1,RHOBL,ASTDL);

C2CDFL=CDF('NORMAL',C2,RHOBL,ASTDL);

ALPHAT=C2CDFL-C1CDFL;END;

C1L=C1;C1U=C1+D;LOOP=0;DALPHA=-10;

DO WHILE(DALPHA<0 | DALPHA>TOLB & LOOP<LOOPM);

C1=(C1L+C1U)/2;

C1CDFU=CDF('NORMAL',C1,RHOBU,ASTDU);

C2=QUANTILE('NORMAL',C1CDFU+ALPHA,RHOBU,ASTDU);

C1CDFL=CDF('NORMAL',C1,RHOBL,ASTDL);

C2CDFL=CDF('NORMAL',C2,RHOBL,ASTDL);

ALPHAT=C2CDFL-C1CDFL;

DALPHA=ALPHAT-ALPHA;LOOP=LOOP+1;

IF DALPHA<0 THEN C1U=C1;ELSE C1L=C1;END;

REAL=C1;REAU=C2;FINISH;

START EFLUFUN;

TOLB=1E-10;D=0.05;

ALPHAT=0;LOOPM=1E03;LOOP1=0;

TAUBL=LOG((1+RHOBL)/(1-RHOBL))/2;

TAUBU=LOG((1+RHOBU)/(1-RHOBU))/2;

TAUVAR=1/(N-3);TAUSTD=SQRT(TAUVAR);

C1=(TAUBL+TAUBU)/2;

DO WHILE(ALPHAT<ALPHA & LOOP1<LOOPM);

C1=C1-D;

C1CDFU=CDF('NORMAL',C1,TAUBU,TAUSTD);

C2=QUANTILE('NORMAL',C1CDFU+ALPHA,TAUBU,TAUSTD);

C1CDFL=CDF('NORMAL',C1,TAUBL,TAUSTD);

C2CDFL=CDF('NORMAL',C2,TAUBL,TAUSTD);

ALPHAT=C2CDFL-C1CDFL;END;

C1L=C1;C1U=C1+D;LOOP=0;DALPHA=-10;

DO WHILE(DALPHA<0 | DALPHA>TOLB & LOOP<LOOPM);

C1=(C1L+C1U)/2;

C1CDFU=CDF('NORMAL',C1,TAUBU,TAUSTD);

C2=QUANTILE('NORMAL',C1CDFU+ALPHA,TAUBU,TAUSTD);

C1CDFL=CDF('NORMAL',C1,TAUBL,TAUSTD);

C2CDFL=CDF('NORMAL',C2,TAUBL,TAUSTD);

ALPHAT=C2CDFL-C1CDFL;

DALPHA=ALPHAT-ALPHA;LOOP=LOOP+1;

IF DALPHA<0 THEN C1U=C1;ELSE C1L=C1;END;

TAUEL=C1;TAUEU=C2;

REFL=(EXP(2#TAUEL)-1)/(EXP(2#TAUEL)+1);

REFU=(EXP(2#TAUEU)-1)/(EXP(2#TAUEU)+1);FINISH;

START EAPOWERFUN;RUN EALUFUN;

AVAR=((1-RHO##2)##2)/(N-3);ASTD=SQRT(AVAR);

ZEAL=(REAL-RHO)/ASTD;ZEAU=(REAU-RHO)/ASTD;

POWEREA=CDF('NORMAL',ZEAU)-CDF('NORMAL',ZEAL);

FINISH;

START EFPOWERFUN;RUN EFLUFUN;

TAURHO=LOG((1+RHO)/(1-RHO))/2;

TAUVAR=1/(N-3);TAUSTD=SQRT(TAUVAR);

ZEFL=(TAUEL-TAURHO)/TAUSTD;ZEFU=(TAUEU-TAURHO)/TAUSTD;

POWEREF=CDF('NORMAL',ZEFU)-CDF('NORMAL',ZEFL);

FINISH;

RUN EAPOWERFUN;RUN EFPOWERFUN;

PRINT 'EQUT-R ' REAL[FORMAT=8.4] REAU[FORMAT=8.4] POWEREA[FORMAT=8.4];

PRINT 'EQUT-ZETA_HAT' REFL[FORMAT=8.4] REFU[FORMAT=8.4] POWEREF[FORMAT=8.4];

QUIT;

FILE S3

SAS/IML program for computing the sample sizes of the correlation equivalence procedures

PROC IML;

*USER SPECIFICATION PORTION;

*TYPE I ERROR;ALPHA=0.05;

*NOMINAL POWER;POWER=0.80;;

*TRUE CORRELATION;RHO=0.1;

*EQUIVALENCE BOUNDS;RHOBL=0;RHOBU=0.2;

*END OF SPECIFICATION PORTION;

ZA=PROBIT(1-ALPHA);

PRINT RHO RHOBL RHOBU ALPHA POWER;

START EALUFUN;

TOLB=1E-10;D=0.05;

ALPHAT=0;LOOPM=1E03;LOOP1=0;

AVARL=((1-RHOBL##2)##2)/(N-3);ASTDL=SQRT(AVARL);

AVARU=((1-RHOBU##2)##2)/(N-3);ASTDU=SQRT(AVARU);

C1=(RHOBL+RHOBU)/2;

DO WHILE(ALPHAT<ALPHA & LOOP1<LOOPM);

C1=C1-D;

C1CDFU=CDF('NORMAL',C1,RHOBU,ASTDU);

C2=QUANTILE('NORMAL',C1CDFU+ALPHA,RHOBU,ASTDU);

C1CDFL=CDF('NORMAL',C1,RHOBL,ASTDL);

C2CDFL=CDF('NORMAL',C2,RHOBL,ASTDL);

ALPHAT=C2CDFL-C1CDFL;END;

C1L=C1;C1U=C1+D;LOOP=0;DALPHA=-10;

DO WHILE(DALPHA<0 | DALPHA>TOLB & LOOP<LOOPM);

C1=(C1L+C1U)/2;

C1CDFU=CDF('NORMAL',C1,RHOBU,ASTDU);

C2=QUANTILE('NORMAL',C1CDFU+ALPHA,RHOBU,ASTDU);

C1CDFL=CDF('NORMAL',C1,RHOBL,ASTDL);

C2CDFL=CDF('NORMAL',C2,RHOBL,ASTDL);

ALPHAT=C2CDFL-C1CDFL;

DALPHA=ALPHAT-ALPHA;LOOP=LOOP+1;

IF DALPHA<0 THEN C1U=C1;ELSE C1L=C1;END;

REAL=C1;REAU=C2;FINISH;

START EFLUFUN;

TOLB=1E-10;D=0.05;

ALPHAT=0;LOOPM=1E03;LOOP1=0;

TAUBL=LOG((1+RHOBL)/(1-RHOBL))/2;

TAUBU=LOG((1+RHOBU)/(1-RHOBU))/2;

TAUVAR=1/(N-3);TAUSTD=SQRT(TAUVAR);

C1=(TAUBL+TAUBU)/2;

DO WHILE(ALPHAT<ALPHA & LOOP1<LOOPM);

C1=C1-D;

C1CDFU=CDF('NORMAL',C1,TAUBU,TAUSTD);

C2=QUANTILE('NORMAL',C1CDFU+ALPHA,TAUBU,TAUSTD);

C1CDFL=CDF('NORMAL',C1,TAUBL,TAUSTD);

C2CDFL=CDF('NORMAL',C2,TAUBL,TAUSTD);

ALPHAT=C2CDFL-C1CDFL;END;

C1L=C1;C1U=C1+D;LOOP=0;DALPHA=-10;

DO WHILE(DALPHA<0 | DALPHA>TOLB & LOOP<LOOPM);

C1=(C1L+C1U)/2;

C1CDFU=CDF('NORMAL',C1,TAUBU,TAUSTD);

C2=QUANTILE('NORMAL',C1CDFU+ALPHA,TAUBU,TAUSTD);

C1CDFL=CDF('NORMAL',C1,TAUBL,TAUSTD);

C2CDFL=CDF('NORMAL',C2,TAUBL,TAUSTD);

ALPHAT=C2CDFL-C1CDFL;

DALPHA=ALPHAT-ALPHA;LOOP=LOOP+1;

IF DALPHA<0 THEN C1U=C1;ELSE C1L=C1;END;

TAUEL=C1;TAUEU=C2;

REFL=(EXP(2#TAUEL)-1)/(EXP(2#TAUEL)+1);

REFU=(EXP(2#TAUEU)-1)/(EXP(2#TAUEU)+1);FINISH;

START EAPOWERFUN;RUN EALUFUN;

AVAR=((1-RHO##2)##2)/(N-3);ASTD=SQRT(AVAR);

ZEAL=(REAL-RHO)/ASTD;ZEAU=(REAU-RHO)/ASTD;

POWEREA=CDF('NORMAL',ZEAU)-CDF('NORMAL',ZEAL);

FINISH;

START EFPOWERFUN;RUN EFLUFUN;

TAURHO=LOG((1+RHO)/(1-RHO))/2;

TAUVAR=1/(N-3);TAUSTD=SQRT(TAUVAR);

ZEFL=(TAUEL-TAURHO)/TAUSTD;ZEFU=(TAUEU-TAURHO)/TAUSTD;

POWEREF=CDF('NORMAL',ZEFU)-CDF('NORMAL',ZEFL);

FINISH;

N=20;POWEREA=0;

DO WHILE (POWEREA<POWER);

N=N+1;RUN EAPOWERFUN;END;

PRINT 'EQUT-R ' N POWEREA[FORMAT=8.4];

N=20;POWEREF=0;

DO WHILE (POWEREF<POWER);

N=N+1;RUN EFPOWERFUN;END;

PRINT 'EQUT-ZETA_HAT' N POWEREF[FORMAT=8.4];

QUIT;
