# Supplementary material for: Improved procedures and computer programs for equivalence assessment of correlation coefficients
Source: PLoS One. 2021 May 28;16(5):e0252323. doi: 10.1371/journal.pone.0252323 (PMC8162672; doi:10.1371/journal.pone.0252323)
Supplement: Attachment — Submitted filename: plos_one_equiv_cor.pdf [file pone.0252323.s003.pdf]

Review of *Improved procedures and computer programs for equivalence assessment of correlation coefficients*

I am very grateful to have had the opportunity to review this paper. The topic is obviously of great value to researchers, especially as researchers learn the perils of using traditional difference-based NHSTs to assess a negligible association. The paper was very well written.

The authors follow up on Goertzen & Cribbie and attempt to address the issue of conservative Type I error rates and power with small sample sizes and/or narrow equivalence intervals. There is no arguing that the strategy works, but the question is whether or not the procedure is justifiable. On what basis is this procedure appropriate? Essentially, the authors are saying that since the procedure is conservative with small N/narrow equivalence bounds that they will just adjust the critical values to fix the problem. However, is the procedure defensible? In repeated measures designs we use adjusted degrees of freedom procedures like Greenhouse-Geisser to fix a problem … but in this situation the problem is that Type I errors are too large. Yuan, Chan and colleagues attempted to "fix" the conservative nature of equivalence-testing based strategies for measurement equivalence, fit, etc. in SEM settings, but it is unclear whether those are appropriate. Sometimes problems (like conservative Type I error rates) arise for a reason. Goertzen & Cribbie show that with small sample sizes it is very likely that a researcher will find correlations of moderate magnitude even when the population correlation is 0. For example, with a sample size of N = 10, they report that approximately 75% of sample correlation values (in absolute value) exceed r = .1 and 40% exceed r = .3. Thus, there is a logical reason for the conservativeness at small sample sizes that relates back to the distribution of *r* with small N. To be clear, I am not saying that this strategy should be abandoned, only that the authors need to provide extensive details outlining why this strategy is appropriate. For example, an identical solution to the problem could be to just inflate α, but that would not be well received.

I think not examining power is a huge issue. The conservative Type I error rates are in fact not a problem at all (who wouldn't want LESS chance of a Type I error). However, we know that these conservative Type I error rates lead to lower power … with low power being the important issue. I recommend evaluating power differences between the different approaches considered in the paper.

Minor Points:

I really like the emphasis on effect sizes at the start of the paper, even though the paper is presenting an NHST based procedure.

The notation for the traditional Fisher's Z test is confusing because in $|Z^*| > z_{\alpha/2}$, $z_{\alpha/2}$ would represent the lower tail. This is further complicated by the statement "$z_{\alpha/2}$ is the upper

100(α/2)-th percentile of the standard normal distribution", because it is clear that 100(α/2) would be the lower tail cutoff (e.g., 100(.05/2) = 2.5).

In the 'Numerical Examples' section, I don't like that the author has tied what is a "meaningful correlation value" to what is commonly observed in the literature. Meaningfulness should not be determined by evaluating what is common, and that is what this section is implying to readers.

I found the language surrounding critical values, critical intervals, nonempty critical intervals, etc. to be a little hard to follow. Since the adjusted critical values are the crux of the paper, please be explicit and consistent with your use of terms to identify these values.
