# Supplementary material for: Improved procedures and computer programs for equivalence assessment of correlation coefficients
Source: PLoS One. 2021 May 28;16(5):e0252323. doi: 10.1371/journal.pone.0252323 (PMC8162672; doi:10.1371/journal.pone.0252323)
Supplement: Attachment — Submitted filename: EQURHO1.PONE2.Response to reviewers.pdf [file pone.0252323.s004.pdf]

PONE-D-21-04114.R1
Report to the Academic Editor

The paper has been revised with several changes to take into account all the comments raised in the first review. For ease of reference, the modified contents in the revised manuscript are highlighted in yellow. The detailed responses to the reviewers are also presented in this report. The major revisions are summarized as follows.

1. As suggested, the description of the TOST procedure and related results in Goertzen and Cribbie [31] has been modified. Specifically, the related discussion is presented as follows (pp. 4-5):

"It is prudent to note that the lack of association examined in Goertzen and Cribbie [31] concerns what sort of strength of association is so small that it should be described as negligible. It is also constructive and more versatile to evaluate whether a target correlation is close enough to any specific magnitude of substantive interest with respect to the designated equivalence boundaries. The simulation results of Goertzen and Cribbie [31] revealed that the TOST method based on the Fisher's transformation has a serious disadvantage in achieving the nominal Type I error rates. However, no analytic examination and technical illustration have been provided in the literature to elucidate the causes of the problematic behavior. A thorough investigation is required to clarify the nature of such deficiency and its implications for equivalence testing. Goertzen and Cribbie [31] suggested that the detection of a lack of association requires substantially large sample sizes. Monte Carlo simulation methods may give a potential solution to sample size calculation. It is of practical importance to derive the power function and then combine a numerical search to determine the optimal sample sizes."

2. Simulation study was also conducted to compare the power performance of the equivalence tests for various model configurations. With the significance level $\alpha = 0.05$, the simulated powers of the EQUT-r, EQUT-$\hat{\xi}$, TOST-r, and TOST-$\hat{\xi}$ tests are computed for 10,000 independent samples. The model configurations of correlation coefficient, reference range, and sample size are chosen as $\rho = 0$ and 0.05, $(-\rho_B, \rho_B) = (-0.1, 0.1)$ and $(-0.2, 0.2)$, and $N = 25$, 50, 100, 200, 300, 400, and 500, respectively. The simulated powers of the combined twenty-eight settings are summarized in Table 4 for the four equivalence procedures. The results show that the two suggested procedures have more power than the other two TOST counterparts. Although the differences between these methods diminish for large sample

sizes, their discrepancy can be substantial for small and moderate sample sizes. In particular, due to the extremely conservative behavior or the degeneration of rejection region of the two TOST methods, the resulting power values are zero for ten cases in Table 4. For example, both TOST-r and TOST-$\hat{\zeta}$ methods give no power when $(-\rho_B, \rho_B) = (-0.1, 0.1)$ for $N \leq 200$, or when $(-\rho_B, \rho_B) = (-0.2, 0.2)$ for $N \leq 50$. These are reported in the manuscript as follows (p. 23):

"Power comparisons

The examination of different equivalence procedures further explicates their power behavior for detecting the lack of association through simulation study. With the significance level $\alpha = 0.05$, the simulated powers of the EQUT-r, EQUT-$\hat{\zeta}$, TOST-r, and TOST-$\hat{\zeta}$ tests are computed for 10,000 independent samples. The model configurations of correlation coefficient, reference range, and sample size are chosen as $\rho = 0$ and 0.05, $(-\rho_B, \rho_B) = (-0.1, 0.1)$ and $(-0.2, 0.2)$, and $N = 25$, 50, 100, 200, 300, 400, and 500, respectively. The simulated powers of the combined twenty-eight settings are summarized in Table 4 for the four equivalence procedures. The results show that the two suggested procedures have more power than the other two TOST counterparts. Although the differences between these methods diminish for large sample sizes, their discrepancy can be substantial for small and moderate sample sizes. In particular, due to the extremely conservative behavior or the degeneration of rejection region of the two TOST methods, the resulting power values are zero for ten cases in Table 4. For example, both TOST-r and TOST-$\hat{\zeta}$ methods give no power when $(-\rho_B, \rho_B) = (-0.1, 0.1)$ for $N \leq 200$, or when $(-\rho_B, \rho_B) = (-0.2, 0.2)$ for $N \leq 50$. In view of these results, the two TOST procedures are not recommended for detecting a lack of association. The rejection regions EQUT-r and EQUT-$\hat{\zeta}$ assure that the proposed equivalence procedures have superior Type I error rate and power performance."

3. Reviewer #1 raised the issue that the suggested equivalence procedure may not maintain the Type I error rate for the extreme case with $\rho = 0$, $\rho_B = 0.01$, and $N = 10$. The asymptotic normal distribution of the sample correlation coefficient is given in Equation 2 that $r \mathrel{\dot\sim} N(\rho, \sigma_r^2)$ where $\sigma_r^2 = (1 - \rho^2)^2/(N - 3)$. When $\rho = 0$ and $N = 10$, it is clear that $P\{-0.01 < r < 0.01\} \doteq P\{-0.0265 < Z < 0.0265\} = 0.0211$ where $Z \sim N(0, 1)$. However, this is not the correct procedure to evaluate the Type I error rate. For the hypotheses of H_0: $\rho \leq -$

0.01 or 0.01 ≤ ρ versus H_1: –0.01 < ρ < 0.01, it follows from the supplemental computer program that the rejection region EQUT-*r* = $\{-\hat{r}_{EQUT} < r < \hat{r}_{EQUT}\}$ given in Equation 11 is of the form EQUT-*r* = {–0.0237 < *r* < 0.0237} at α = 0.05. Using the large sample approximation, the nominal Type I error rate for $\rho = \rho_B = 0.01$ and *N* = 10 is *P*{–0.0237 < *r* < 0.0237} $\doteq$ *P*{–0.08917 < *Z* < 0.03625} = 0.0500. Moreover, the accuracy of the rejection region EQUT-*r* = {–0.0237 < *r* < 0.0237} in attaining the specified alpha is also confirmed with a simulation study with 10,000 independent data sets. The simulated Type I error rate is 0.0536 which is rather close to the nominal level 0.05.

4. The simulation results of Goertzen and Cribbie [31] revealed that the TOST method based on the Fisher's transformation has a serious disadvantage in achieving the nominal Type I error rates. Despite the undesirable behavior of the TOST extension for correlation evaluations, no technical examinations and proper alternatives have been described in the literature. The discussion about the empty set rejection region of the TOST method not only clarify TOST's conservative property, but also motives the need for developing a more desirable approach. Essentially, it is of practical value to emphasize that the TOST method for assessing the lack of association does not share the same desirable properties of the counterpart TOST for establishing mean equivalence.

The description of effect size measures provides a brief introduction about the importance of correlation coefficient as a useful effect size index. The second reviewer found the emphasis on effect size is informative. It also justifies the extensive researches that focus on correlation coefficients.

PONE-D-21-04114.R1
Report to Reviewer #1

The paper has been revised with several changes to take into account all the comments raised in the first review. For ease of reference, the modified contents in the revised manuscript are highlighted in yellow. The responses to your comments are presented as follows.

1. A desirable property of a test procedure should adequately control its Type I error rate at the nominal level. This is a fundamental characteristic for examining a statistical test because a test procedure can be liberal or conservative under different model configurations. Throughout this revision, the description has been rephrased about the conservative TOST methods by emphasizing the true Type I error rates can be much less than the specified value. For example, it is noted (p. 5):

"The simulation results of Goertzen and Cribbie [31] revealed that the TOST method based on the Fisher's transformation has a serious disadvantage in achieving the nominal Type I error rates."

2. As suggested, there appears to be a lack of consensus in the literature on which method is most appropriate for equivalence testing. The following description is presented in this revision (p. 29):

"It is essential to note that there is little consensus in the literature on which method is most appropriate for equivalence testing. Conceptually, the preference varies with the right and proper criteria to select an optimal procedure. Considerations of more advanced aspects of TOST and alternative procedures for bioequivalence testing are beyond the scope of this article. The interested reader is referred to Meyners [24], Berger and Hsu [30], and the discussion therein for further details."

3. The suggested equivalence procedure still maintains the Type I error rate for the extreme case with $\rho = 0$, $\rho_B = 0.01$, and $N = 10$. The asymptotic normal distribution of the sample correlation coefficient is given in Equation 2 that $r \mathrel{\dot\sim} N(\rho, \sigma_r^2)$ where $\sigma_r^2 = (1 - \rho^2)^2/(N - 3)$. When $\rho = 0$ and $N = 10$, it is clear that $P\{-0.01 < r < 0.01\} \doteq P\{-0.0265 < Z < 0.0265\}$

= 0.0211 where $Z \sim N(0, 1)$. However, this is not the correct procedure to evaluate the Type I error rate. For the hypotheses of H_0: $\rho \le -0.01$ or $0.01 \le \rho$ versus H_1: $-0.01 < \rho < 0.01$, it follows from the supplemental computer program that the rejection region EQUT-r = $\{-\hat{r}_{EQUT} < r < \hat{r}_{EQUT}\}$ given in Equation 11 is of the form EQUT-r = $\{-0.0237 < r < 0.0237\}$ at $\alpha = 0.05$. Using the large sample approximation, the nominal Type I error rate for $\rho = \rho_B = 0.01$ and $N = 10$ is $P\{-0.0237 < r < 0.0237\} \doteq P\{-0.08917 < Z < 0.03625\}$ = 0.0500. Moreover, the accuracy of the rejection region EQUT-r = $\{-0.0237 < r < 0.0237\}$ in attaining the specified alpha is also confirmed with a simulation study with 10,000 independent data sets. The simulated Type I error rate is 0.0536 which is rather close to the nominal level 0.05.

Details

1. The simulation results of Goertzen and Cribbie [31] revealed that the TOST method based on the Fisher's transformation has a serious disadvantage in achieving the nominal Type I error rates. Despite the undesirable behavior of the TOST extension for correlation evaluations, no technical examinations and proper alternatives have been described in the literature. The discussion about the empty set rejection region of the TOST method not only clarify TOST's conservative property, but also motives the need for developing a more desirable approach. Essentially, it is of practical value to emphasize that the TOST method for assessing the lack of association does not share the same desirable properties of the counterpart TOST for establishing mean equivalence.

The description of effect size measures provides a brief introduction about the importance of correlation coefficient as a useful effect size index. The second reviewer found the emphasis on effect size is informative. It also justifies the extensive researches that focus on correlation coefficients.

2. Specific chapters are provided about the properties of sample correlation coefficient in the texts of Johnson et al. [11] and Stuart and Ord [12] as follows (p. 3):

"Theoretical details and related issues can be found in Chapter 32 of Johnson et al. [11] and Chapter 16 of Stuart and Ord [12]."

PONE-D-21-04114.R1
Report to Reviewer #2

The paper has been revised with several changes to take into account all the comments raised in the first review. For ease of reference, the modified contents in the revised manuscript are highlighted in yellow. The responses to your comments are presented as follows.

1. Regarding the justification of the proposed equivalence approaches, it is noted in the manuscript (p. 8) that the general theorem to deriving optimal parametric tests for equivalence hypotheses was presented in Wellek [27], Section 3.3. Also, the determination of rejection region of the optimal procedure follows from the general results in Lehmann and Romano [38], Section 3.4, for tests in families with monotone likelihood ratio. The proposed extensions for equivalence assessments of correlations are asymptotically optimal because the results rely on the large-sample normal approximations. In addition to the theoretical arguments, the statistical properties of Type I error control and power performance for the suggested equivalence methods are also illustrated in the numerical investigations. On the other hand, the conservative behavior of the TOST methods with small sample sizes and narrow equivalence bounds suggests that they cannot be accepted as a general tool for making sound application.

2. The results in Table 1 of Goertzen and Cribbie [31] revealed that it would be extremely difficult to prove a lack of association when sample sizes are small to moderate. Specifically, with a sample size of $N = 10$, approximately 75% of sample correlation values (in absolute value) exceed $r = 0.1$, 40% exceed $r = 0.3$, and even 15% exceed $r = 0.5$. Accordingly, the conventional tests of difference are inappropriate for assessing an effect is absent based a non-significant result. Notably, the recommended approach is to conduct an equivalence test to ascertain whether the observed correlation falls inside the selected equivalence boundaries.

3. As suggested, simulation study was also conducted to compare the power performance of the equivalence tests for various model configurations. With the significance level $\alpha = 0.05$, the simulated powers of the EQUT-r, EQUT-$\hat{\zeta}$, TOST-r, and TOST-$\hat{\zeta}$ tests are computed for 10,000 independent samples. The model configurations of correlation

coefficient, reference range, and sample size are chosen as $\rho = 0$ and 0.05, $(-\rho_B, \rho_B) = (-0.1, 0.1)$ and $(-0.2, 0.2)$, and $N = 25, 50, 100, 200, 300, 400$, and 500, respectively. The simulated powers of the combined twenty-eight settings are summarized in Table 4 for the four equivalence procedures. The results show that the two suggested procedures have more power than the other two TOST counterparts. Although the differences between these methods diminish for large sample sizes, their discrepancy can be substantial for small and moderate sample sizes. In particular, due to the extremely conservative behavior or the degeneration of rejection region of the two TOST methods, the resulting power values are zero for ten cases in Table 4. For example, both TOST-r and TOST-$\hat{\zeta}$ methods give no power when $(-\rho_B, \rho_B) = (-0.1, 0.1)$ for $N \le 200$, or when $(-\rho_B, \rho_B) = (-0.2, 0.2)$ for $N \le 50$.

These are reported in the manuscript as follows (p. 23):

"Power comparisons

The examination of different equivalence procedures further explicates their power behavior for detecting the lack of association through simulation study. With the significance level $\alpha = 0.05$, the simulated powers of the EQUT-r, EQUT-$\hat{\zeta}$, TOST-r, and TOST-$\hat{\zeta}$ tests are computed for 10,000 independent samples. The model configurations of correlation coefficient, reference range, and sample size are chosen as $\rho = 0$ and 0.05, $(-\rho_B, \rho_B) = (-0.1, 0.1)$ and $(-0.2, 0.2)$, and $N = 25, 50, 100, 200, 300, 400$, and 500, respectively. The simulated powers of the combined twenty-eight settings are summarized in Table 4 for the four equivalence procedures. The results show that the two suggested procedures have more power than the other two TOST counterparts. Although the differences between these methods diminish for large sample sizes, their discrepancy can be substantial for small and moderate sample sizes. In particular, due to the extremely conservative behavior or the degeneration of rejection region of the two TOST methods, the resulting power values are zero for ten cases in Table 4. For example, both TOST-r and TOST-$\hat{\zeta}$ methods give no power when $(-\rho_B, \rho_B) = (-0.1, 0.1)$ for $N \le 200$, or when $(-\rho_B, \rho_B) = (-0.2, 0.2)$ for $N \le 50$. In view of these results, the two TOST procedures are not recommended for detecting a lack of association. The rejection regions EQUT-r and EQUT-$\hat{\zeta}$ assure that the proposed equivalence procedures have superior Type I error rate and power performance."

Minor points

1. As in Goertzen and Cribbie [31] and other related references, the particular notation $z_{\alpha/2}$ represents the upper 100(α/2)-th percentile of the standard normal distribution. Accordingly, the lower 100(α/2)-th percentile of the standard normal distribution can be simplified as $-z_{\alpha/2}$ because the standard normal distribution is symmetric. Although such notation is not unified in the literature, the related terms are carefully defined without any ambiguity.

2. The statement has been rephrased as “target effects” (p. 25):
“A research study requires adequate statistical power and sufficient sample size to examine vital questions and target effects.”

3. All the terms of “critical interval” and “critical region” are rephrased as “rejection region” in this revision.
